# Pathogenic variant burden in the ExAC database: an empirical approach to evaluating population data for clinical variant interpretation

**DOI:** 10.1186/s13073-017-0403-7

**Published:** 2017-02-06

**Authors:** Yuya Kobayashi, Shan Yang, Keith Nykamp, John Garcia, Stephen E. Lincoln, Scott E. Topper

**Affiliations:** Invitae Corporation, 1400 16th St., San Francisco, CA 94103 USA

**Keywords:** Allele-frequency threshold, Variant interpretation, ExAC, ACMG ISV guidelines

## Abstract

**Background:**

The frequency of a variant in the general population is a key criterion used in the clinical interpretation of sequence variants. With certain exceptions, such as founder mutations, the rarity of a variant is a prerequisite for pathogenicity. However, defining the threshold at which a variant should be considered “too common” is challenging and therefore diagnostic laboratories have typically set conservative allele frequency thresholds.

**Methods:**

Recent publications of large population sequencing data, such as the Exome Aggregation Consortium (ExAC) database, provide an opportunity to characterize with accuracy and precision the frequency distributions of very rare disease-causing alleles. Allele frequencies of pathogenic variants in ClinVar, as well as variants expected to be pathogenic through the nonsense-mediated decay (NMD) pathway, were analyzed to study the burden of pathogenic variants in 79 genes of clinical importance.

**Results:**

Of 1364 *BRCA1* and *BRCA2* variants that are well characterized as pathogenic or that are expected to lead to NMD, 1350 variants had an allele frequency of less than 0.0025%. The remaining 14 variants were previously published founder mutations. Importantly, we observed no difference in the distributions of pathogenic variants expected to be lead to NMD compared to those that are not. Therefore, we expanded the analysis to examine the distributions of NMD expected variants in 77 additional genes. These 77 genes were selected to represent a broad set of clinical areas, modes of inheritance, and penetrance. Among these variants, most (97.3%) had an allele frequency of less than 0.01%. Furthermore, pathogenic variants with allele frequencies greater than 0.01% were well characterized in publications and included many founder mutations.

**Conclusions:**

The observations made in this study suggest that, with certain caveats, a very low allele frequency threshold can be adopted to more accurately interpret sequence variants.

**Electronic supplementary material:**

The online version of this article (doi:10.1186/s13073-017-0403-7) contains supplementary material, which is available to authorized users.

## Background

With the increasing adoption of whole-genome, exome, and panel-based genetic testing, the detection of novel, previously uncharacterized sequence variants has increased dramatically. Currently, approximately 85% of sequence variants in ClinVar have been reported only by single submitters and, despite growth in both the number of ClinVar participants and total entries, this percentage has remained steady [1]. We have undoubtedly entered an era in which detection of variants far outpaces the ability of researchers to gather genetic data or generate experimental data to assess potential phenotypic consequences. With such limited data, more than 40% of the variants in ClinVar are still designated as variants of uncertain significance.

One class of empirical data, however, has great potential for improving variant interpretation: population allele frequency data. According to the joint consensus recommendation for the interpretation of sequence variants by the American College of Medical Genetics and Genomics (ACMG) and the Association for Molecular Pathology (AMP), an “allele frequency greater than expected for disorder” is strong evidence for a benign classification [2]. However, the recommendation provides no detailed guidance for determining the expected allele frequency of pathogenic variants. Furthermore, most population databases do not provide phenotypic information on individuals included and are rarely a collection of individuals unburdened with disease. Understanding and accounting for this fact is critical in determining the frequency threshold at which a variant can be considered “greater than expected for disorder.” After the release of the Exome Variant Server (EVS) dataset, Norton et al. [3] and Shearer et al. [4] proposed and explored strategies for using the allele frequency of known pathogenic variants to determine a disease-specific MAF threshold. These approaches were powerful in supporting a dramatic reduction in MAF thresholds for two diseases; however, their applications are limited to genes for which high-quality curated lists of pathogenic variants are available. Furthermore, the usefulness of such data for clinical variant interpretation was limited because allele frequencies were calculated using small datasets, resulting in frequency estimates of limited accuracy.

**Fig. 1:**
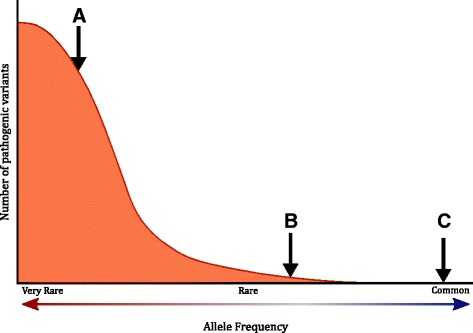
Concept diagram for using pathogenic variant frequency distributions to establish allele frequency thresholds. Depicted is a density plot of pathogenic variants in a hypothetical gene. *x-axis*: allele frequency; *y-axis*: number of pathogenic variants. The *arrows* (labeled A, B, and C) highlight three different scenarios of how allele frequencies of previously uncharacterized variants can be evaluated in the context of the pathogenic variant frequency distribution. In scenario A, the uncharacterized variant has an allele frequency that is highly consistent with known pathogenic variants. Because many benign variants are also rare or private, the allele frequency of this variant provide little weight towards either classification. In scenario B, the uncharacterized variant has an allele frequency that is consistent with known pathogenic variants, but is more common than the vast majority of them. The likelihood of such a variant being pathogenic is substantially reduced. In scenario C, the uncharacterized variant has an allele frequency outside of the pathogenic variant frequency distribution. The likelihood of such a variant being pathogenic is extremely low, as it is more common than any other previously characterized pathogenic variant

This situation changed significantly with the publication of the Exome Aggregation Consortium (ExAC) population dataset comprising exome sequencing data from 60,706 unrelated individuals [5]—a nearly tenfold increase in the data compared to previously available population databases. This dramatic increase in cohort size results in a more comprehensive representation of very rare variants and allows for more accurate minor allele frequency (MAF) calculations. These advantages allow for the direct and accurate characterization of the population burden of pathogenic variants associated with rare Mendelian disorders [5–8]. Such characterizations, in turn, can be used to measure the likelihood of whether a novel, previously uncharacterized variant is too common to be consistent with what is expected of pathogenic variants in this cohort (Fig. 1).

In this study, we sought to develop a method for determining MAF thresholds that does not rely on comprehensive, curated lists of pathogenic variants. To do so, we began by exploring the population burden of representative pathogenic variants in the ExAC cohort. Specifically, we examined the distributions of known pathogenic variants in *BRCA1* and *BRCA2* (*BRCA1/2*)—well-characterized genes with loss-of-function molecular mechanisms—and showed that the frequency of pathogenic variants in the ExAC dataset is very low, does not depend on variant type, and is consistent with disease incidence in the general population. We then applied an analysis of the frequency of premature-termination variants to 77 other genes, including genes associated with hereditary cancer, genes with autosomal recessive inheritance patterns, genes with substantially reduced penetrance, and to a set of genes that are generally less well studied. This empirical approach to identifying the frequency characteristics of pathogenic variants supports the use of ExAC to calculate of MAF thresholds that are substantially lower than current industry norms for a broad set of genes.

## Methods

### ExAC data processing

Version 0.3 of the ExAC dataset was used as a data source in this study [9]. Only variants with the ExAC bioinformatics filter status of “PASS” were included in the analysis. Furthermore, to ensure adequate population depth for accurate allele frequency calculations, we restricted our analyses to loci with a minimum cohort depth of 80,000 total alleles (AN_Adj). The “AC_Adj” data points were used for allele counts.

To identify variants expected to lead to nonsense-mediated decay (NMD_positive_), we started with variants designated by ExAC as “HC” (high-confidence) loss-of-function (nonsense, frameshift and consensus splice site variants). We further filtered variants to exclude those that are not expected to be subject to NMD; specifically, a variant was removed if the predicted premature-termination codon occurred in the final exon, within 50 bp of the final exon − exon junction, or as part of the consensus splice site dinucleotide in the final intron [10]. Also removed were variants with published experimental data indicating that they escaped NMD. Additionally, analyses were restricted to NMD predictions relative to clinically relevant transcripts. Clinically relevant transcripts were determined based on reviews of the Human Genome Mutation Database (HGMD) [11] and published literature (see Additional file 4). Variants were designated as NMD_negative_ if they do not meet the criteria for NMD_positive_. These include: missense variants, synonymous variants, intronic variants beyond the consensus splice site dinucleotides, in-frame indels, and truncating variants that are not expected to lead to NMD.

### ClinVar data processing

The January 2016 ClinVar Full Release XML file was used as a data source in this study [12]. To avoid dubious variant classification calls, we restricted the analyses to interpretations from a subset of the submitters (see Additional file 5).

For the evaluation of pathogenic NMD_negative_ variants in *BRCA1/2*, variants were included if they had a unanimous consensus classification of pathogenic or likely pathogenic from multiple submitters. NMD_negative_ variants classified pathogenic or likely pathogenic by a single submitter or lacking consensus among multiple submitters were evaluated based on the 2015 ACMG guidelines for the interpretation of sequence variants [4]. Variants were included if the pathogenic or likely pathogenic classification was attained with this method.

Variant classifications from HGMD were not considered in this, as many disease-associated variants in that dataset have been shown not to be causative [13–15].

### Identifying published literature for variants

To identify a comprehensive list of publications for a given variant, searches were performed with several data sources and search tools including HGMD, ClinVar, SETH, NCBI PubTator, and Google. Variants described at the DNA and protein levels, with considerations for both legacy naming and HGVS nomenclature, were used as search terms.

## Results

Historically, small cohorts of presumed healthy individuals have been used to distinguish benign polymorphisms from potentially pathogenic variants. This approach is effective for early-onset dominant disorders with high penetrance, as any variants observed in unaffected individuals are unlikely to be disease causing. By contrast, modern population datasets such as ExAC and EVS are typically aggregates of multiple large-scale sequencing projects and often include individuals recruited for disease-specific studies [3, 5]. Therefore, the likelihood is high that disease-causing variants are present in these cohorts. This likelihood increases even further when one considers low-penetrance, late-onset, or recessive disorders in which unaffected carriers are expected to be present. The clinical utility of large population databases in variant interpretation is contingent on accounting for the presence of these variants. To this end, we first evaluated the prevalence of pathogenic variants in ExAC for a number of well-studied genes.

### Allele frequency distributions of pathogenic variants in *BRCA1/2*

We first evaluated the frequency of pathogenic variants in *BRCA1/2*, genes that are known to cause hereditary breast and ovarian cancer (HBOC) [16, 17]. *BRCA1/2* are among the most well studied and clinically tested genes and there is little disagreement about the pathogenicity of most variants [18]. These characteristics gave us high confidence that the catalog of observed pathogenic variants is nearly comprehensive for recurrent variants and largely uncontroversial. We expect to find many unaffected carriers in the general population because the prevalence of HBOC is relatively high [19, 20], the disease has an adult onset, and pathogenic *BRCA1/2* variants are incompletely penetrant [21, 22], particularly in male carriers. Finally, because more than one-tenth of the ExAC population was derived from The Cancer Genome Atlas (TCGA) patient cohort [5], the ExAC dataset might contain an enrichment of pathogenic variants compared to the general population.

**Fig. 2:**
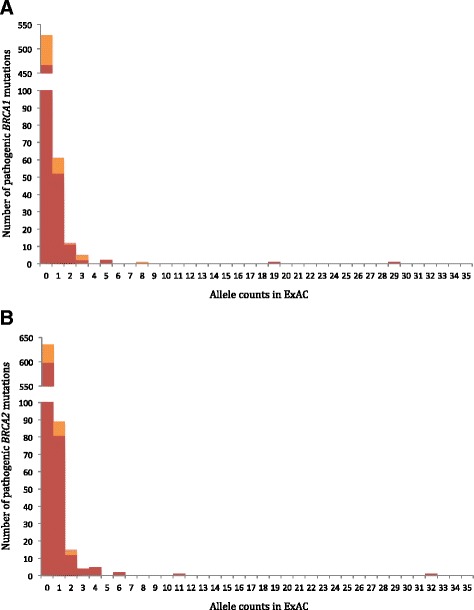
Allele counts of *BRCA1* and *BRCA2* pathogenic and likely pathogenic variants. Histograms of the allele counts of pathogenic and likely pathogenic variants in the ExAC dataset for (**a**) *BRCA1* and (**b**) *BRCA2. x-axis*: allele count; *y-axis*: number of unique sequence variants. The *red* portion represents pathogenic NMD_positive_ variants and the *orange* portion represents pathogenic NMD_negative_ variants (missense, intronic, in-frame indels, and truncations expected to avoid NMD)

In total, there were 592 *BRCA1* and 712 *BRCA2* unique pathogenic variants in ClinVar. An additional 60 variants that were present in ExAC, but not in ClinVar, and are expected to be pathogenic based on variant type, were also included in the analysis. These variants included nonsense or frameshift variants that are sufficiently 5′ in the gene to be expected to subject the messenger RNA transcript to NMD [10], and therefore loss of protein expression, and variants that are part of the consensus splice donor or acceptor dinucleotides [23]. These types of variants were collectively designated as NMD_positive_ variants, while those that are not expected to lead to NMD were designated as NMD_negative_ variants. The vast majority of pathogenic variants were extremely rare: of the total 1364 pathogenic variants, 1163 (85.3%) were absent from the ExAC cohort and an additional 150 (11.0%) were each observed in ExAC in a single individual (Fig. 2). All 60 of the non-ClinVar NMD_positive_ variants found in ExAC were among those observed only in single individuals (Additional file 1). From the perspective of allele frequencies, 1356/1364 (99.4%) of the pathogenic variants had allele frequencies of less than 0.005%. The difference in the frequencies of pathogenic variants compared to frequencies of all BRCA1/2 variants in ExAC was statistically significant (*p* < 8.5E-11; Wilcoxon rank-sum test).

ExAC also contains 14 pathogenic variants observed in more than three individuals. All of these have been reported as population-specific founder mutations or have been otherwise well characterized in the literature as common recurrent variants (Table 1). Excluding these variants, the next most common variant was present at a frequency of 0.0025%. The most common founder mutation, *BRCA2* c.5946delT (p.Ser1982Argfs*22) [24], was present at a frequency of 0.027%.

**Table 1:** Most common pathogenic *BRCA1* and *BRCA2* variants in ExAC

Gene	Variant	Effect	Allele count	MAF	Type
*BRCA1*	NM_007294.3:c.68_69delAG	p.Glu23Valfs*17	29	0.024%	Founder [24]
*BRCA1*	NM_007294.3:c.5266dupC	p.Gln1756Profs*74	19	0.016%	Founder [24]
*BRCA1*	NM_007294.3:c.181 T > G	p.Cys61Gly	8	0.0067%	Founder [36]
*BRCA1*	NM_007294.3:c.4035delA	p.Glu1346Lysfs*20	5	0.0041%	Founder [37]
*BRCA1*	NM_007294.3:c.1687C > T	p.Gln563*	5	0.0041%	Founder [38]
*BRCA2*	NM_000059.3:c.5946delT	p.Ser1982Argfs*22	32	0.027%	Founder [24]
*BRCA2*	NM_000059.3:c.3847_3848delGT	p.Val1283Lysfs*2	11	0.011%	Recurrent [39]
*BRCA2*	NM_000059.3:c.658_659delGT	p.Val220Ilefs*4	6	0.0061%	Recurrent [40, 41]
*BRCA2*	NM_000059.3:c.7480C > T	p.Arg2494*	6	0.0050%	Founder [42]
*BRCA2*	NM_000059.3:c.3545_3546delTT	p.Phe1182*	4	0.0033%	Recurrent [43, 44]
*BRCA2*	NM_000059.3:c.3599_3600delGT	p.Cys1200*	4	0.0033%	Recurrent [45]
*BRCA2*	NM_000059.3:c.5576_5579delTTAA	p.Ile1859Lysfs*3	4	0.0033%	Recurrent [46, 47]
*BRCA2*	NM_000059.3:c.7069_7070delCT	p.Leu2357Valfs*2	4	0.0033%	Recurrent [48]
*BRCA2*	NM_000059.3:c.9118-2A > G	Splice acceptor	4	0.0033%	Recurrent [39, 49]

Fourteen pathogenic variants in *BRCA1* and *BRCA2* were observed in more than three individuals in the ExAC cohort. All of these variants have been previously reported as founder or common recurrent variants. Some of these recurrent variants are suggested to be founder mutations, but have not been confirmed as such. *MAF* minor allele frequency

We compared the frequency distributions of pathogenic NMD_positive_ variants to that of pathogenic NMD_negative_ variants and found no statistically significant difference: Wilcoxon rank-sum test failed to reject the null hypothesis that the distributions of NMD_positive_ and NMD_negative_ pathogenic variants are equal (*p* = 0.72). A few missense variants in *BRCA1* had previously been characterized as being hypomorphic [25], which raised the possibility that they might have been present at allele frequencies higher than those of null variants. This does not appear to be the case, however. For the two putatively hypomorphic missense variants examined, p.Ser1497Ala was absent in the ExAC cohort and p.Arg1699Gln was observed in three individuals. One missense variant, p.Cys61Gly, that was previously reported as a founder mutation in multiple ethnic groups was observed in eight individuals [25]. These observations suggest that an analysis of pathogenic NMD_positive_ variants can provide conclusions representative of all variant types.

### Allele frequency distributions of NMD_positive_ variants in other clinically relevant genes

In the analysis of *BRCA1/2* variants we noted that there was no apparent enrichment for observations of pathogenic NMD_negative_ variants compared with pathogenic NMD_positive_ variants and that even known hypomorphic missense variants were present at very low allele frequencies. These observations suggest that for genes with loss-of-function mechanisms of disease, a pathogenic allele frequency distribution determined by only NMD_positive_ variants can serve as a close approximation of the comprehensive set of pathogenic variants (Additional file 1). Based on this observation, we expanded our analysis to several dozen clinically relevant genes across multiple clinical areas where the disease mechanism has been established as loss of function (Table 2).

**Table 2:** Frequencies of NMD_positive_ variants in hereditary cancer, primary ciliary dyskinesia (PCD), and arrhythmia/cardiomyopathy genes in ExAC

		Variant allele frequencies in ExAC:				
Gene	Inheritance	<0.005%	0.005–0.01%	0.01–0.05%	0.05–0.1%	≥0.1%
Hereditary cancer						
*APC*	Dominant	6	-	-	-	-
*ATM*	Recessive	104	1	-	-	-
*BARD1*	Dominant	22	-	-	-	-
*BLM*	Recessive	38	2	1	-	-
*BMPR1A*	Dominant	3	1	-	-	-
*BRCA1*	Dominant	67	-	2	-	-
*BRCA2*	Dominant	103	2	1	-	-
*BRIP1*	Recessive	32	-	1	-	-
*CDH1*	Dominant	5	-	-	-	-
*CDKN2A*	Dominant	4	-	-	-	-
*CHEK2*	Dominant	29	3	2	-	1
*CTRC*	Dominant	11	1	-	-	-
*EPCAM*	Recessive	9	1	-	-	-
*FANCC*	Recessive	23	1	-	-	-
*MEN1*	Dominant	-	-	-	-	-
*MLH1*	Dominant	5	-	-	-	-
*MRE11A*	Recessive	18	1	-	-	-
*MSH2*	Dominant	7	1	-	-	-
*MSH6*	Dominant	25	-	1	-	-
*MUTY*	Recessive	19	3	2	-	1
*NBN*	Recessive	33	1	1	-	-
*NF1*	Dominant	23	1	-	-	-
*PALB2*	Dominant	35	2	1	-	-
*PMS2*	Dominant	32	-	-	-	-
*PTCH1*	Dominant	2	-	-	-	-
*PTEN*	Dominant	2	-	-	-	-
*RAD50*	Recessive	42	2	3	-	-
*RAD51C*	Recessive	19	3	-	-	-
*SMAD4*	Dominant	2	-	-	-	-
*SPINK1*	Dominant	1	-	1	-	-
*STK11*	Dominant	-	-	-	-	-
*TP53*	Dominant	1	-	-	-	-
*VHL*	Dominant	-	-	-	-	-
Primary ciliary dyskinesia						
*ARMC4*	Recessive	36	3	-	-	-
*C21orf59*	Recessive	7	1	1	-	-
*CCDC103*	Recessive	3	-	-	-	-
*CCDC114*	Recessive	6	-	-	-	-
*CCDC151*	Recessive	16	1	-	-	-
*CCDC39*	Recessive	22	2	-	-	-
*CCDC40*	Recessive	32	1	1	-	-
*CCDC65*	Recessive	17	-	1	-	-
*CCNO*	Recessive	-	-	-	-	-
*DNAAF1*	Recessive	19	2	1	-	-
*DNAAF2*	Recessive	12	-	-	-	-
*DNAAF3*	Recessive	16	2	1	-	-
*DNAH11*	Recessive	80	1	2	-	-
*DNAH5*	Recessive	113	-	2	-	-
*DNAI1*	Recessive	15	-	1	-	-
*DNAI2*	Recessive	21	1	1	-	-
*DNAL1*	Recessive	3	-	-	-	-
*DRC1*	Recessive	22	-	2	-	-
*DYX1C1*	Recessive	18	1	-	-	-
*RPGR*	Recessive	1	-	-	-	-
*RSPH1*	Recessive	9	1	2	-	-
*RSPH4A*	Recessive	27	1	-	-	-
*RSPH9*	Recessive	4	-	-	-	-
*SPAG1*	Recessive	21	2	-	-	-
*ZMYND10*	Recessive	16	-	-	-	-
Arrhythmia and cardiomyopathy						
*BAG3*	Dominant	2	-	-	-	-
*CACNA1C*	Dominant	6	-	-	-	-
*CASQ2*	Recessive	15	-	-	-	-
*DES*	Recessive	5	-	-	-	-
*DSC2*	Dominant	13	-	-	-	-
*DSG2*	Dominant	19	-	-	-	-
*DSP*	Recessive	21	-	-	-	-
*FHL1*	Dominant	-	-	-	-	-
*HCN4*	Dominant	5	-	-	-	-
*JUP*	Recessive	7	-	-	-	-
*KCNE1*	Dominant	-	-	-	-	-
*KCNH2*	Dominant	5	-	2	-	-
*KCNQ1*	Dominant	15	-	1	-	-
*LAMP2*	Dominant	-	-	-	-	-
*LMNA*	Dominant	1	-	-	-	-
*MYBPC3*	Dominant	17	-	-	-	-
*NKX2-5*	Dominant	1	-	-	-	-
*PKP2*	Dominant	18	-	1	-	-
*PLN*	Dominant	-	-	-	-	-
*SCN5A*	Dominant	9	-	-	-	-
*TRDN*	Recessive	6	1	2	-	-

All NMD_positive_ variants for genes associated with hereditary cancer, primary ciliary dyskinesia, and arrhythmia/cardiomyopathy were binned based on their allele frequencies in the ExAC dataset. In all genes listed, loss of protein function has been established as the mechanism of disease. Several of the genes listed are associated with both dominant and recessive inheritance patterns; the listed inheritance patterns are specifically for those associated with the disorders most relevant to the clinical area, and the mechanism of disease is loss of function. For example, *BRCA2* is associated with autosomal dominant hereditary breast and ovarian cancer, as well as autosomal recessive Fanconi anemia. *DES* and *DSP* genes are associated with both dominant and recessive arrhythmia and cardiomyopathy disorders. However, the loss-of-function mechanism has only been firmly established with the recessive disorders

We initially examined 31 additional genes associated with hereditary cancer syndromes. These genes are also associated with adult-onset conditions with incomplete penetrance. Consistent with the observations in *BRCA1*/*2*, NMD_positive_ variants in these genes were rare, and the vast majority (576/591) had allele frequencies of less than 0.01%. Only two variants were reported at allele frequencies greater than 0.05%: *CHEK2* c.1100delC, a well-characterized Northern European founder mutation [26]; and *MUTYH* c.934-2A > G, a splice-site variant previously reported to have a high carrier frequency in individuals of East Asian descent [27]. Including these two variants, 15 total variants had allele frequencies greater than 0.01%, of which eight have been reported as founder or suspected founder mutations.

Next, we examined genes suspected for various reasons to harbor higher frequencies of pathogenic variants in the ExAC database. We analyzed 25 genes that cause primary ciliary dyskinesia (PCD), as these genes have a recessive mode of inheritance and the presence of unaffected heterozygous carriers are likely to result in higher allele frequencies of pathogenic variants. In addition, because the single largest cohort within ExAC was derived from the Myocardial Infarction Genetics Consortium [5]—which may result in enrichment for pathogenic variants in cardiology-related genes—we analyzed 21 genes known to cause arrhythmias and cardiomyopathies through loss-of-function mechanisms. Among the 742 NMD_positive_ variants across these 46 genes, none reached an allele frequency greater than 0.05%. For the PCD genes, seven of the 15 NMD_positive_ variants with allele frequencies greater than 0.01% were previously published as founder or suspected founder mutations. For the genes associated with arrhythmias and cardiomyopathies, only six NMD_positive_ variants had allele frequencies greater than 0.01%, one of which is a known founder mutation (Additional file 2). Interestingly, NMD_positive_ variants in five gain-of-function cardiomyopathy and arrhythmia genes were also very rare with 93 of 96 such variants being present at less than 0.005% frequency. This is consistent with the understanding that even benign variants are often very rare. Strikingly, however, the ABCC9 gene contained two NMD_positive_ variants that were present in greater than 0.01%: c.565C > T (p.Arg189*) at 0.013% and c.2238-1G > A at 0.16% (Additional file 3).

Because there are many other cardiomyopathy-associated and arrhythmia-associated genes in which the disease mechanism is gain-of-function, this analysis alone cannot eliminate the possibility that the ExAC cohort is enriched for individuals with pathogenic NMD_negative_ variants in those genes. To examine this possibility further, we analyzed previously classified missense variants. However, compared with *BRCA1*/*2*, these genes accounted for substantially fewer submissions to ClinVar. Therefore, we referred to a previously published “gold standard” set of 74 missense variants from six genes associated with hypertrophic cardiomyopathy [28]. This set, which was published before the release of the ExAC dataset, includes 41 pathogenic missense variants and 33 benign or likely benign missense variants (Table 3). Of the pathogenic missense variants, 29 were not observed in the ExAC cohort. The most common pathogenic missense variant was *MYH7* c.2389G > A (p.Ala797Thr), which was observed with an allele frequency of 0.0033%. The *MYBPC3* variant c.1504C > T (p.Arg502Trp) is considered one of the most common pathogenic variants of hypertrophic cardiomyopathy in individuals of European descent [29] and it was observed with an allele frequency of 0.0025%.

**Table 3:** ExAC allele frequencies of the 74 hypertrophic cardiomyopathy “gold standard” missense variants

Gene	Variant	Effect	Classification	MAF
*MYBPC3*	NM_000256.3:c.772G > A	p.Glu258Lys	Pathogenic	0.0039%^a^
*MYH7*	NM_000257.2:c.2389G > A	p.Ala797Thr	Pathogenic	0.0033%
*MYBPC3*	NM_000256.3:c.1504C > T	p.Arg502Trp	Pathogenic	0.0025%
*MYH7*	NM_000257.2:c.2167C > T	p.Arg723Cys	Pathogenic	0.0025%
*TNNI3*	NM_000363.4:c.485G > A	p.Arg162Gln	Pathogenic	0.0025%
*MYH7*	NM_000257.2:c.1988G > A	p.Arg663His	Pathogenic	0.0016%
*MYBPC3*	NM_000256.3:c.1484G > A	p.Arg495Gln	Pathogenic	0.00083%
*MYL2*	NM_000432.3:c.173G > A	p.Arg58Gln	Pathogenic	0.00083%
*MYL2*	NM_000432.3:c.64G > A	p.Glu22Lys	Pathogenic	0.00083%
*TNNI3*	NM_000363.4:c.433C > T	p.Arg145Trp	Pathogenic	0.00083%
*MYH7*	NM_000257.2:c.2609G > A	p.Arg870His	Pathogenic	0.00082%^b^
*TNNT2*	NM_001001430.1:c.274C > T	p.Arg92Trp	Pathogenic	0.00082%
*TNNI3*	NM_000363.4:c.433C > G	p.Arg145Gly	Pathogenic	0.00080%
*MYBPC3*	NM_000256.3:c.1351G > C	p.Glu451Gln	Pathogenic	0
*MYBPC3*	NM_000256.3:c.1505G > A	p.Arg502Gln	Pathogenic	0
*MYBPC3*	NM_000256.3:c.2265C > A	p.Asn755Lys	Pathogenic	0
*MYH7*	NM_000257.2:c.1207C > T	p.Arg403Trp	Pathogenic	0
*MYH7*	NM_000257.2:c.1208G > A	p.Arg403Gln	Pathogenic	0
*MYH7*	NM_000257.2:c.1357C > T	p.Arg453Cys	Pathogenic	0
*MYH7*	NM_000257.2:c.1750G > C	p.Gly584Arg	Pathogenic	0
*MYH7*	NM_000257.2:c.1816G > A	p.Val606Met	Pathogenic	0
*MYH7*	NM_000257.2:c.2146G > A	p.Gly716Arg	Pathogenic	0
*MYH7*	NM_000257.2:c.2155C > T	p.Arg719Trp	Pathogenic	0
*MYH7*	NM_000257.2:c.2156G > A	p.Arg719Gln	Pathogenic	0
*MYH7*	NM_000257.2:c.2167C > G	p.Arg723Gly	Pathogenic	0
*MYH7*	NM_000257.2:c.2221G > T	p.Gly741Trp	Pathogenic	0
*MYH7*	NM_000257.2:c.2717A > G	p.Asp906Gly	Pathogenic	0
*MYH7*	NM_000257.2:c.2722C > G	p.Leu908Val	Pathogenic	0
*MYH7*	NM_000257.2:c.2770G > A	p.Glu924Lys	Pathogenic	0
*MYH7*	NM_000257.2:c.2788G > A	p.Glu930Lys	Pathogenic	0
*MYH7*	NM_000257.2:c.4135G > A	p.Ala1379Thr	Pathogenic	0
*MYH7*	NM_000257.2:c.438G > T	p.Lys146Asn	Pathogenic	0
*MYH7*	NM_000257.2:c.767G > A	p.Gly256Glu	Pathogenic	0
*TNNI3*	NM_000363.4:c.470C > T	p.Ala157Val	Pathogenic	0
*TNNI3*	NM_000363.4:c.557G > A	p.Arg186Gln	Pathogenic	0
*TNNI3*	NM_000363.4:c.575G > A	p.Arg192His	Pathogenic	0
*TNNT2*	NM_001001430.1:c.236 T > A	p.Ile79Asn	Pathogenic	0
*TNNT2*	NM_001001430.1:c.275G > A	p.Arg92Gln	Pathogenic	0
*TNNT2*	NM_001001430.1:c.421C > T	p.Arg141Trp	Pathogenic	0
*TPM1*	NM_000366.5:c.523G > A	p.Asp175Asn	Pathogenic	0
*TPM1*	NM_000366.5:c.688G > A	p.Asp230Asn	Pathogenic	0
*MYBPC3*	NM_000256.3:c.2686G > A	p.Val896Met	Likely benign	1.28%^a^
*MYBPC3*	NM_000256.3:c.833G > A	p.Gly278Glu	Likely benign	0.29%^a^
*TNNI3*	NM_000363.4:c.244C > T	p.Pro82Ser	Likely benign	0.28%
*MYBPC3*	NM_000256.3:c.565G > A	p.Val189Ile	Likely benign	0.27%
*MYBPC3*	NM_000256.3:c.3413G > A	p.Arg1138His	Likely benign	0.13%
*MYBPC3*	NM_000256.3:c.1519G > A	p.Gly507Arg	Likely benign	0.068%
*MYBPC3*	NM_000256.3:c.3004C > T	p.Arg1002Trp	Likely benign	0.067%
*TNNI3*	NM_000363.4:c.235C > T	p.Arg79Cys	Likely benign	0.043%
*MYBPC3*	NM_000256.3:c.1564G > A	p.Ala522Thr	Likely benign	0.039%
*MYBPC3*	NM_000256.3:c.440C > T	p.Pro147Leu	Likely benign	0.038%^a^
*MYH7*	NM_000257.2:c.3981C > A	p.Asn1327Lys	Likely benign	0.010%
*MYBPC3*	NM_000256.3:c.1147C > G	p.Leu383Val	Likely benign	0.0089%
*MYBPC3*	NM_000256.3:c.842G > A	p.Arg281Gln	Likely benign	0.0070%^a^
*MYBPC3*	NM_000256.3:c.1633C > A	p.Leu545Met	Likely benign	0.0034%
*MYBPC3*	NM_000256.3:c.1246G > A	p.Gly416Ser	Likely benign	0.0029%
*MYBPC3*	NM_000256.3:c.2063C > A	p.Thr688Lys	Likely benign	0.0019%^a^
*MYBPC3*	NM_000256.3:c.3142C > T	p.Arg1048Cys	Likely benign	0.0017%
*TNNT2*	NM_001001430.1:c.805A > T	p.Asn269Tyr	Likely benign	0.0017%^a^
*MYBPC3*	NM_000256.3:c.2410C > A	p.Leu804Met	Likely benign	0
*MYH7*	NM_000257.2:c.321 T > G	p.Asp107Glu	Likely benign	0
*MYH7*	NM_000257.2:c.4555A > T	p.Ser1519Cys	Likely benign	0
*MYH7*	NM_000257.2:c.8A > C	p.Asp3Ala	Likely benign	0
*TNNI3*	NM_000363.4:c.244C > A	p.Pro82Thr	Likely benign	0
*TNNI3*	NM_000363.4:c.253 T > A	p.Leu85Met	Likely benign	0
*TNNI3*	NM_000363.4:c.257C > A	p.Ala86Asp	Likely benign	0
*TNNT2*	NM_001001430.1:c.682C > G	p.Gln228Glu	Likely benign	0
*MYBPC3*	NM_000256.3:c.706A > G	p.Ser236Gly	Benign	10.79%
*MYBPC3*	NM_000256.3:c.472G > A	p.Val158Met	Benign	9.04%^a^
*TNNT2*	NM_001001430.1:c.758A > G	p.Lys253Arg	Benign	5.07%
*MYH7*	NM_000257.2:c.4472C > G	p.Ser1491Cys	Benign	0.75%
*MYBPC3*	NM_000256.3:c.977G > A	p.Arg326Gln	Benign	0.55%
*MYBPC3*	NM_000256.3:c.1144C > T	p.Arg382Trp	Benign	0.42%
*MYBPC3*	NM_000256.3:c.2498C > T	p.Ala833Val	Benign	0.22%

Jordan et al. [28] previously published a curated list of 74 “gold standard” missense variants in genes associated with hypertrophic cardiomyopathy. All pathogenic variants were observed at allele frequencies of less than 0.005% in the ExAC dataset

^a^Loci covered by less than 80,000 total alleles in ExAC

^b^A variant that did not pass the variant calling quality filter in ExAC

Two common themes emerged from our analysis of all of the genes we evaluated: (1) the vast majority of pathogenic variants were extremely rare (allele frequency less than 0.01%); and (2) variants with allele frequencies above 0.01% were generally already well characterized in the literature. We speculate that these outliers reached elevated allele frequencies owing to mutational hot spot effects or population history, such as bottleneck events. Consistent with this speculation, no correlation was observed between the number of outliers or the allele frequencies of outliers and disease severity, penetrance, or inheritance pattern.

### Accounting for outlier-frequency pathogenic variants through literature review

Without careful consideration, aggressive allele frequency thresholds may increase the risk of incorrectly classifying pathogenic variants with elevated allele frequencies as benign. However, this problem is neither unique nor new, as many in the clinical genetics community currently adopt thresholds lower than the MAFs of many founder mutations. Well-known examples include two *BRCA1/2* variants observed at MAFs of 1% in the Ashkenazi Jewish population (*BRCA1* c.68_69delAG and *BRCA2* c.5946_5949delTGGA) [24] and the *CFTR* ΔF508 variant observed at 1% frequency in the European population [30, 31]. Because these variants are by definition frequently observed, they are typically accompanied by many published studies that support a pathogenic classification despite the greater than expected frequency.

**Fig. 3:**
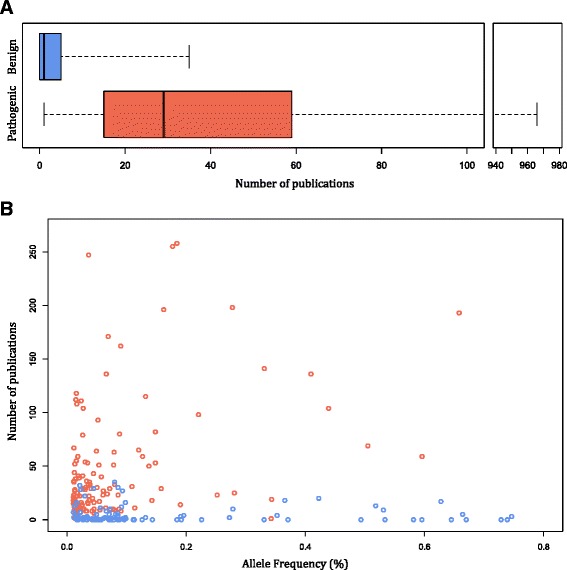
Number of publications for variants observed at an allele frequency greater than 0.01%. **a** The top *box plot*, in *blue*, summarizes the publication of a randomly selected set of 100 variants with a consensus classification of benign in ClinVar and having an allele frequency greater than 0.01%. The bottom *box plot*, in *red*, summarizes the publication counts of all 129 variants with a consensus classification of pathogenic in ClinVar and having an allele frequency greater than 0.01%. The box represents the 25th–75th percentile range, with the median publication count depicted as the horizontal line in the middle. The “*whiskers*” represents the maximum and minimum values. **b** *Scatter plot* of allele frequencies and publication counts of the same set of variants. Each *red circle* represents a pathogenic variant and each *blue circle* represents a benign variant. *x-axis*: allele frequency; *y-axis*: publication counts. Four pathogenic variants that are extreme outliers were excluded for display purposes: (1) GJB2 NM_004004.5:c.35delG (p.Gly12Valfs*2) has an allele frequency of 0.60% and reported in 496 publications; (2) CFTR NM_000492.3:c.1521_1523delCTT (p.Phe508del) has an allele frequency of 0.68% and reported in 966 publications; (3) SERPINA1 NM_001127701.1:c.1096G > A (p.Glu366Lys) has an allele frequency of 1.2% and reported in 56 publications; and (4) BTD NM_000060.3:c.1330G > C (p.Asp444His) has an allele frequency of 3.2% and reported in 46 publications

To assess whether a thorough literature review would be sufficient to identify outlier-frequency pathogenic variants, we measured publication counts for this type of variant. Specifically, variants with allele frequencies greater than 0.01% and ClinVar consensus classifications of pathogenic (two or more submitters all agreeing on designations of pathogenic or likely pathogenic) were evaluated. Among all of the entries in ClinVar—spanning thousands of genes—only 129 variants from 79 genes met these criteria and each of these 129 consensus pathogenic variants had been reported in at least one published study (Fig. 3a). The median value was 29 publications and 20 of these variants were described in more than 100 publications. For comparison, the analysis was repeated for a selection of 100 variants with a ClinVar consensus classification of benign. This set was deliberately restricted to match the allele frequency range of the 129 consensus pathogenic variants but was otherwise randomly chosen. Among these benign variants, 47 had zero publications and three-fourths had fewer than five publications. For pathogenic variants, a statistically significant but weak correlation was found between allele frequency and publication counts (r = 0.24, *p* < 0.0063), whereas no correlation was observed for benign variants (r = –0.03) (Fig. 3b). This difference is the result of a striking enrichment of publications among pathogenic variants compared with benign variants (*p* < 2.2E-16; Wilcoxon rank-sum test). These data suggest that pathogenic variants as rare as 0.01% are routinely observed and published and that the scientific literature can and should be used in concert with population data for variant classification.

## Discussion

Traditional methods for setting allele frequency thresholds for variant classification are based on the expected incidence of disease [4]. However, except for a handful of extremely well-studied and relatively common diseases, accurate incidence and penetrance numbers simply do not exist. Furthermore, the numbers that are available are undermined by selection bias, uncertainty about the phenotypic heterogeneity derived from pathogenic variants in the gene, uncertainty about how many different pathogenic alleles account for the total incidence of disease, and for many rare diseases, the relative paucity of identified cases. Meaningful calculations cannot be performed with inaccurate constants.

In this study, we suggest a bottom-up approach—defining allele frequency thresholds through an empirical analysis of the burden of pathogenic variants in a particular dataset—rather than the theoretical approach of beginning with an assessment of disease incidence and penetrance. We evaluated the pathogenic variant load in ExAC on a gene-by-gene basis to determine the thresholds at which a variant might be considered to be either “within the pathogenic range” or where they might be considered extremely unlikely to be pathogenic. In all of the evaluated genes, which spanned multiple clinical areas, levels of penetrance, ages of onset, rates of disease prevalence and modes of inheritance, we found that the vast majority of NMD_positive_ variants were extremely rare, with 97.3% of them being observed with MAFs of less than 0.01% in the ExAC cohort. We also observed that pathogenic NMD_negative_ variants displayed equivalent frequency distributions. Therefore, by relying on NMD_positive_ variants as a representative subset of all pathogenic variants, we were able to apply this approach to a broad set of genes regardless of how many variants had been published in the public domain. This result is consistent with our understanding that these diseases are rare and typically caused by many individually rare or private variants rather than a few common variants [32, 33].

However, because of its reliance on NMD_positive_ variants, this gene-by-gene examination is restricted to diseases for which the disease mechanism has been confirmed to be loss of function. As we encountered with hypertrophic cardiomyopathy, this limitation adds challenges for diseases with gain-of-function mechanisms. Moreover, although setting gene-specific allele frequency thresholds is theoretically possible, such an approach is likely impractical in a clinical laboratory setting where hundreds or thousands of genes are simultaneously evaluated. As such, a hybrid approach may be warranted when the types of observations made in this study are combined with our, albeit limited, understanding of disease incidence and penetrance. In this study, we deliberately examined sets of genes expected to have relatively high observations of pathogenic variants in the ExAC cohort, whether due to high disease incidence, low penetrance, recessive conditions, or potential enrichment in the ExAC cohort. Therefore, an allele frequency threshold derived from this set of genes can be considered the “upper-bound” for all other genes with lower incidences and/or higher penetrance.

The presence of founder mutations remains a concern in any approach based on population frequency, including the method presented herein; however, the risk is mitigated by the well-studied nature of most of the subpopulations in the ExAC database. Indeed, all pathogenic variants we examined that had MAFs greater than 0.01% have been previously reported in the published literature. As such, comprehensive literature reviews of variants at these higher MAF ranges still remain an essential component of variant classification. While this is not a significant hurdle for targeted testing approaches in which literature review is routine, it is an important consideration for whole-genome and whole-exome studies in which variants are often filtered by MAF. Of note, benign variants in the same MAF range had on average 12-fold fewer publications and nearly half of these variants have never been reported in the literature. This suggests that the necessary burden of reviewing literature on these benign variants is minimal.

For this study, we did not consider ethnic subpopulation data. This was a deliberate decision based on our initial observation that most pathogenic variants are extremely rare in the overall global population. Given the large variability in the cohort sizes of various ethnic groups represented in ExAC, subdividing the frequency data would have added complexities that were extraneous to the aims of this study. However, subpopulation-specific allele frequency data remain invaluable tools for identifying population-specific polymorphisms as well as putative founder mutations. Indeed, recent publications have successfully used ExAC subpopulation data to identify cases of genetic misdiagnosis; these variants were previously classified as pathogenic, but are now believed to be benign polymorphisms that are overrepresented in certain ethnic groups [7, 34].

The pathogenic allele frequency distributions described in this study are specific to the ExAC cohort, but the approach is generalizable. The allele frequencies of variants are a function of the fitness burden of the variant as well as any skewing that results from non-random selection of the cohort, which can include factors such as ethnic composition or potential enrichment for particular diseases based on study design. These would all be considered confounding factors in traditional approaches to MAF threshold calculations. However, because our method measures allele frequency distributions directly, these factors are no longer significant concerns; this is the key advantage of this approach compared with calculations based simply on disease incidence and penetrance. The implication of this direct approach is that MAF thresholds can and should be reconsidered for each population dataset. This is a scalable approach that allows for rapid adoption of new datasets and refinements to MAF thresholds as larger and higher-quality datasets are published.

Analyzing the burden of genetic variation in and of itself is not a novel concept. Previous works that measured residual variation intolerance scores (RVIS) and probability of being loss-of-function intolerant (pLI) played important roles in identifying genes of clinical importance [5, 35]. By examining the ratio of observed and expected number of genetic variations, these approaches gave clinical geneticists the ability to detect signatures of selection on a gene-by-gene basis: genes with less observed variation than expected are likely to be subject to purifying selection. The study presented in this manuscript is, in spirit, a continuation of those works. Here, we evaluated the observed genetic variation, which is the outcome of selection occurring on disease-causing variants, to answer the question of what variant frequency is sufficient to designate the variant as too common to cause disease.

Finally, this study was almost entirely dependent on shared data (ExAC and ClinVar). As researchers and clinical laboratories share more data and work collaboratively, we anticipate that the collective knowledge and experiences will greatly improve the community’s ability to classify variants accurately, which will in turn lead to better patient care.

## Conclusions

In clinical genetics, variant classification is a complex process involving the evaluation and interpretation of multiple pieces of evidence, which in turn requires considerable knowledge and expertise. A variant’s absence from ExAC or presence in ExAC at very low frequency is clearly not sufficient to indicate that the variant is pathogenic. Many variants are private, novel, or rare, and the vast majority of these are also not pathogenic. However, with certain clear exceptions such as founder mutations, the rarity of the variant is a prerequisite for pathogenicity.

As mentioned above, in the joint consensus recommendation for the interpretation of sequence variants by the ACMG and AMP, it is stated that an “allele frequency greater than expected for disorder” should be considered strong evidence for a benign classification. Based on the observations made in this study, global ExAC allele frequencies greater than 0.01% should be considered “greater than expected” for diseases of Mendelian inheritance and this threshold may be lowered even further for certain genes such as *BRCA1* and *BRCA2*. Ultimately, however, a benign or likely benign classification should be made in the context of allele frequency, reports in the published literature, and the confidence of the underlying data. The method outlined in this study is intended to assist clinical geneticists in better evaluating and using large population datasets.

## Additional files

Additional file 1: Figure S1. Histograms of the allele counts of NMD_positive_ variants in the ExAC dataset for (**A**) *BRCA1* and (**B**) *BRCA2. x-axis*: allele count; *y-axis*: number of unique sequence variants. The *solid* portion represents variants that have been reported in ClinVar, and the *shaded* portion represents those that are absent from ClinVar. (PNG 40 kb)
Additional file 2: Table S1. List of the all NMD_positive_ variants with allele frequencies greater than 0.01% in the set of genes described in Table 2. Known and suspected founder mutations are indicated. *PCD* primary ciliary dyskinesia. (XLSX 48 kb)
Additional file 3: Table S2. Frequencies of NMD_positive_ variants in gain-of-function arrhythmia/cardiomyopathy genes in ExAC. (XLSX 31 kb)
Additional file 4: Table S3. List of clinically relevant transcripts for hereditary cancer, PCD, and cardiology genes. For each gene examined, a single clinically relevant transcript was selected to make NMD predictions. *PCD* primary ciliary dyskinesia. (XLSX 47 kb)
Additional file 5: Table S4. List of ClinVar submitters. The analysis presented in this work were based on ClinVar entries from the listed submitters only. The submitter names are as they appear in the ClinVar XML file and may include multiple names from the same institution. (XLSX 9 kb)

## Abbreviations

ACMG: American College of Medical Genetics and Genomics
AMP: Association for Molecular Pathology
ExAC: Exome Aggregation Consortium
EVS: Exome Variant Server
HBOC: Hereditary breast and ovarian cancer
HGMD: Human Genome Mutation Database
MAF: Minor allele frequency
NMD: Nonsense-mediated decay
PCD: Primary ciliary dyskinesia
pLI: Probability of being loss-of-function intolerant
RVIS: Residual variation intolerance scores
TCGA: The Cancer Genome Atlas
