# Supplementary figures and images for: Pathogenic variant burden in the ExAC database: an empirical approach to evaluating population data for clinical variant interpretation
Source: Genome Med. 2017 Feb 6;9:13. doi: 10.1186/s13073-017-0403-7 (PMC5295186; doi:10.1186/s13073-017-0403-7)

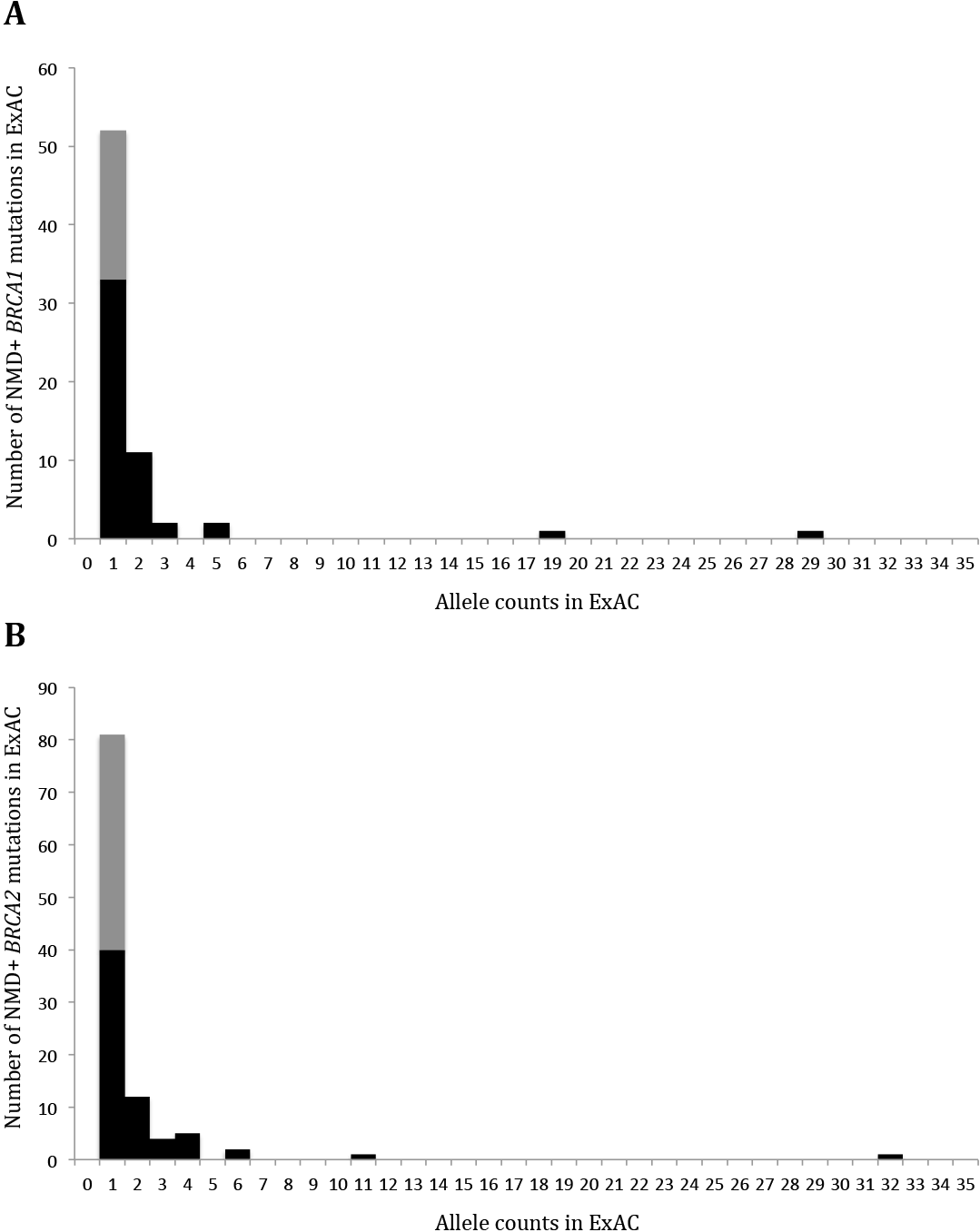

Supplement: Additional file 1: Figure S1. — Histograms of the allele counts of NMDpositive variants in the ExAC dataset for (A) BRCA1 and (B) BRCA2. x-axis: allele count; y-axis: number of unique sequence variants. The solid portion represents variants that have been reported in ClinVar, and the shaded portion represents those that are absent from ClinVar. (PNG 40 kb) [file 13073_2017_403_MOESM1_ESM.png]
